# Israeli registered nurse workforce

**DOI:** 10.1186/2045-4015-1-12

**Published:** 2012-03-12

**Authors:** Greer Glazer

**Affiliations:** 1 College of Nursing, University of Cincinnati College of Nursing, 3110 Vine Street, 416 Procter Hall, PO Box 210038, Cincinnati, Ohio 45221-0038, USA

**Keywords:** Nursing workforce, nursing supply, Israeli nursing workforce

## Abstract

This commentary on the article by Nirel, Riba, Reicher and Toren, "Registered nurses in Israel - workforce employment characteristics and projected supply", describes major findings from this important Israeli study and links findings to other nursing workforce studies worldwide. Israeli projections include a 25% decrease in RNs in the workforce by 2028; the greater likelihood of leaving the progression of young nurses compared to older nurses, and the greater likelihood of leaving the profession by those nurses with no advanced training. Suggestions are made for future workforce analysis to triangulate economic analysis and policy planning, work design, and workforce management; for policy and planning and budget allocation at the macro level to go hand-in-hand with work design and management strategies at the micro level; and for the development of a national nursing workforce plan for investment and reform with a timeline and specific dates for accomplishing separate goals for recruitment and retention.

This is a commentary on http://www.ijhpr.org/content/1/1/11/.

## Commentary

The purpose of the study by Nirel, Riba, Reicher and Toren [1] was to provide an in-depth review of the existing supply of Israel's nursing workforce: job and employment characteristics, internal and external mobility, working lifespan, and the projected supply in view of exits and future entry sources. Nursing workforce studies are critically important to individual countries as well as the international nursing community since there are nursing shortages across all Westernized healthcare systems. The World Health Organization (WHO) identifies 57 countries with severe nursing shortages capable of affecting the delivery of essential nursing care [2]. Shortages of nurses, the largest group of healthcare providers in the healthcare workforce, will negatively impact individual and population health. More RN hours with patients are related to decreased mortality rates, decubiti, nosocomial infections, cardiac and respiratory failure, pneumonia, and failure to rescue [3-5]. Nursing shortages also negatively impact practicing nurses by increasing workload, increasing stress, decreasing morale and satisfaction, and creating burnout, resulting in intent to leave or exit from the profession.

The major reasons for the nursing shortages worldwide are similar. They are: 1) aging of the workforce, 2) changes in the work environment/climate, 3) decreased or insufficient enrollment in nursing education programs, and 4) poor image of nursing. While the supply of nurses is decreasing worldwide, the demand for nursing services is increasing and will continue to escalate due to the aging of the population, increasing health disparities, the obesity epidemic, and a dramatic increase in people with one or more chronic diseases. In fact, the U.S. population 65 years and older is expected to double by 2030 [6]; in addition, approximately 133 million Americans have at least one chronic illness [7].

Major findings from this important Israeli study are the projected 25% decrease of RNs in the workforce, from 28,500 in 2008 to 21,201 in 2028; the significantly greater likelihood of leaving the nursing profession of young (24-34-year-old) nurses compared to nurses who are 34-44 years old (5 times) and 45-62 years old (4 times); and the three times greater likelihood of leaving the nursing profession by those nurses with no advanced training.

Projections of the future size of the Israeli nursing workforce depend, in part, on the recent and projected trends in the number of people entering the profession. This study recognized that the numbers will decrease based on the projected decline of Former Soviet Union immigrant practical nurses retrained as RNs, and the Israeli policy decision to discontinue practical nursing tracks. Projections of the future size of the workforce also depend on retention of nurses. Others addressing nurse retention projections have analyzed how long nurses themselves expect to stay in the profession in the future, researchers and analysts' projections of how long nurses will stay in the profession in the future, and work climate/environment factors that affect how long nurses stay in the profession. The projected decrease in RN workforce in Israel may be underestimated in this study by Nirel, Riba, Reicher and Toren because they have chosen to base their projections on survival rates, an analysis of how long nurses have stayed in the profession in the past. The operational definition of the survival rate variable, the likelihood of leaving work in the profession, had two components: time (duration of work as an RN) and occurrence (quitting or continuing work in the profession). The survival rates (nurses remaining in the profession) in this random sample were 97% after 5 years, 93% after 10 years, and 88% after 20 years. These rates are higher than rates posited in other countries that have been based on likelihood of staying in the profession, and included work climate/environment factors. Milisen et al. [8] reported that 54% of Belgian nurses have contemplated leaving at some point in time. None of the work climate/environment variables, nor the image of nursing in Israel, were included in this study. The two major reasons nurses leave the profession are not feeling valued (lack of respect, recognition, and autonomy) and the belief that they cannot deliver high quality care (due to workload, poor staffing, lack of organizational support, and safety issues). Other work climate/environment factors include work-related injuries (more than 40% of nurses have back, neck, or buttock pain some to all of the time [9]), bullying (more than 25% in most studies), and greater choice for women in other professions. The U.S. National Sample Survey of Registered Nurses [10] reported that approximately 466,564 RNs were not employed in nursing in 2008. Forty-one percent of unemployed RNs under 50 years old were not working in nursing due to workplace problems such as stressful work environment, poor management, and burnout. Israeli registered nurses may stay in the profession less than they have in the past due to negative work climate/environment factors and the image of nursing that is dominated by medicine and physicians. Future nursing workforce supply studies should include these work climate/environment variables in estimating survival rate and likelihood to leave the profession.

The finding that younger nurses are more likely than older nurses to leave the nursing profession is consistent with other studies and an Israeli study [11] that found younger nurses were less satisfied than older nurses with the profession. Policy makers should develop a range of multigenerational strategies [12] for retaining nurses, as well as attracting and motivating nurses of all generations, since each generation has its own set of values, worldview, attitudes about work and authority, communication patterns, and expectations about work/life balance.

The importance of providing access to advanced education for nurses is highlighted by the threefold increase in leaving the nursing profession for those nurses without advanced education. Progressive increased Finance Ministry support for advanced nursing education through 2028 may be key to retention of nurses in the Israeli workforce. Implementation of a professional development model similar to one used in the U.S. where nurses spent 20% of their time on professional development, resulting in reduced turnover and greater staff satisfaction [13], might also reduce the number of nurses leaving the profession.

Current and future workforce analysis should triangulate economic analysis and policy planning, work design, and workforce management [8]. Policy and planning and budget allocation at the macro level need to go hand-in-hand with work design and management strategies at the micro level. There should be a focus on investments in work environment redesign that allows nurses to provide comprehensive, quality, professional nursing care. Examination of the position of professional nursing within the Israeli healthcare system would shed light on a major variable related to projected supply and intent to leave the profession. Nurse migration within and between countries is projected to grow and is one of the defining issues of this century [14]. Policy makers and key stakeholders should develop a national nursing workforce plan for investment and reform with a timeline and specific dates for accomplishing separate goals for recruitment and retention. Specific targets should be set for number of nurses graduating from RN programs, retention of current nurses, and the projected contribution of foreign-educated nurses to their in-country nursing workforce. Rarely mentioned, setting targets for ethnically, racially, and religiously diverse registered nurses to mirror the Israeli population would be a major addition to a national workforce plan. Identification of the responsible parties for implementation of all strategies must be clear. This RN workforce study and suggested future studies will contribute to "having the right people with the right skills in the right place at the right time to provide the right services to the right people" [[15], p. 109]. The key to a future Israeli nursing workforce that will meet the needs of the public will be to get the workforce research used for decision-making in policy and practice.

## Competing interests

The author declares that they have no competing interests.

## Authors' information

Greer Glazer is the Dean and Schmidlapp Professor of Nursing at the College of Nursing at the University of Cincinnati.

## References

[B1] Nirel N Riba S Reicher S Toren O Registered nurses in Israel - workforce employment characteristics and projected supply Isr J of Health Policy Res 2012 1 11 10.1186/2045-4015-1-11 PMC3424825 22913612

[B2] World Health Organization The World Heath Report 2006: Working Together for Health 2006 Geneva, Switzerland: WHO

[B3] Aiken LH Sloane DM Lake ET Sochalski J Weber AL Organization and outcomes of inpatient AIDS care Med Care 1999 37 760 762 10.1097/00005650-199908000-00006 10448719

[B4] Buerhaus PI Staiger DO Auerbach DI Is the current shortage of hospital nurses ending? Health Affair 2003 22 191 198 10.1377/hlthaff.22.6.191 14649446

[B5] Mark BA Harless DW McCue M Xu Y A longitudinal examination of hospital registered nurse staffing and quality of care Health Serv Res 2004 39 279 300 10.1111/j.1475-6773.2004.00228.x 15032955 PMC1361008

[B6] Josiah Macy Jr. Foundation Conference Summary Ensuring an Effective Physician Workforce for the United States: Recommendations for Reforming Graduate Medical Education to meet the needs of the public 2011 http://josiahmacyfoundation.org/docs/macy_pubs/Macy_GME_Report,_Aug_2011.pdf

[B7] Guroff M Diagnosis: Love AARP The Magazine 2011 55 1A 36 41 56

[B8] Milisen K Abraham I Siebens K Darras E Dierckx de Casterlé B BELIMAGE group Work environment and workforce problems: a cross-sectional questionnaire survey of hospital nurses in Belgium Int J Nurs Stud 2006 43 745 754 10.1016/j.ijnurstu.2005.10.008 16321387

[B9] O'Brien-Pallas L Hayes L Challenges in getting workforce research in nursing used for decision-making in policy and practice: a Canadian perspective J Clin Nurs 2008 17 3338 3346 10.1111/j.1365-2702.2008.02641.x 19146593

[B10] U.S. Department of Health and Human Services, Health Resources and Services Administration The Registered Nurse Population findings from the 2008 National Sample Survey of Registered Nurses 2010 Washington, DC: HRSA

[B11] Nirel N Yair Y Samuel H Riba S Reicher S Toren O Registered Nurses in Israel: Workforce Supply - Patterns and Trends 2010 Jerusalem: Myers-JDC-Brookdale Institute RR-557-10

[B12] Stanley D Multigenerational workforce issues and their implications for leadership in nursing J Nurs Manage 2010 18 846 852 10.1111/j.1365-2834.2010.01158.x 20946220

[B13] Bournes DA Ferguson-Paré M Human becoming and 80/20: an innovative professional development model for nurses Nurs Sci Q 2007 20 237 253 10.1177/0894318407303126 17595405

[B14] Nichols B Davis C Richardson D Mason D, Leavitt J, Coffee M Global nurse migration Policy and Politics in Nursing and Health Care 2011 St. Louis: Elsevier Saunders 401 408

[B15] Birch S Health human resource planning for the new millennium: inputs in the production of health, illness and recovery in populations Can J Nurs Res 2002 33 109 114 11998188

